# Nephrotic syndrome due to focal segmental glomerulosclerosis complicating scleroderma: a case report

**DOI:** 10.1186/s13256-023-04273-6

**Published:** 2024-01-15

**Authors:** Mahsa Mehdipour Dalivand, Asghar Hadjiabbasi, Elham Ramezanzadeh, Seyed Mahmood Habibzadeh, Kimia Goudarzi, Reza Shahriarirad, Habib Zayeni

**Affiliations:** 1 Rheumatology Research Center, Department of Internal Medicine, School of Medicine, Shariati Hospital, Tehran University of Medical Sciences, Tehran, Iran; 2 Guilan Rheumatology Research Center, Department of Rheumatology, Razi Hospital, School of Medicine, Guilan University of Medical Sciences, Rasht, Iran; 3 Guilan Nephrology Research Center, Department of Nephrology, Razi Hospital, School of Medicine, Guilan University of Medical Sciences, Rasht, Iran; 4 Razi Private Laboratory, Rasht, Gilan Iran; 5 School of Medicine, Shiraz University of Medical Sciences, Shiraz, Iran

**Keywords:** Focal segmental glomerulosclerosis, Nephrotic syndrome, Pathology, Systemic scleroderma

## Abstract

**Background:**

Systemic scleroderma (SSc) is an insidious autoimmune connective tissue disorder with multiorgan involvement. Renal involvement is one of the important causes of morbidity and mortality in scleroderma; however, nephrotic syndrome is reported rarely in association with SSc. We present a patient with SSc who developed focal segmental glomerulosclerosis (FSGS) as a complication of scleroderma.

**Case presentation:**

A 59 year old Caucasian female patient, with a known history of diffuse systemic sclerosis from 8 years, presented to our clinic with symptoms of anasarca and weight gain. Her physical examination was unremarkable except for periorbital and extremity edema. Her biochemistry assessment revealed decreased serum albumin levels and elevated serum creatinine levels. A renal biopsy was performed, which showed histopathological patterns of FSGS type of nephrotic syndrome. After administration of high doses of steroid and rituximab in the course of her treatment for 6 months, her symptoms and proteinuria were improved without the occurrence of scleroderma renal crises.

**Conclusion:**

SSc is a complex multisystemic autoimmune disorder. SRC is the most prominent renal involvement in SSc, but other renal pathologies may also occur. Each patient should be precisely investigated since managing these renal conditions can differ significantly. Nephrotic syndrome is a rare complication of SSc, which could be managed with prompt diagnosis and steroid administration.

## Introduction

Systemic sclerosis (SSc) is a multisystemic chronic connective tissue disorder resulting from dysregulation in innate and adaptive immune systems, leading to chronic inflammation, skin and many organs fibrosis, and vasculopathy [1]. Multiple organs, including skin, lungs, gastrointestinal tract, heart, kidneys, and musculoskeletal system, are targeted in this disease [2], and depending on the extent and pattern of skin involvement, SSc is categorized into two subtypes of limited and diffuse SSc which have different complications as well [3]. Individuals diagnosed with limited cutaneous SSc (lcSSc) typically experience skin involvement distal to the elbows or knees, which may or may not involve the facial area, while patients with diffuse cutaneous SSc (DcSSc) exhibit skin changes that extend proximally to their elbows or knees. The onset of the disease in LcSSc is often gradual, but these patients are more likely to have digital ulcers or pulmonary arterial hypertension. On the other hand, patients with DcSSc have a rapid onset of skin changes with internal organ involvement. Due to the severity of the disease in patients with DsSSc, they have a 10-year survival rate of 65%, compared to 92% for those with LcSSc [4].

Renal involvement prevalence in SSc patients was approximately 50%; moreover, autopsy evaluation of patients diagnosed with SSc showed occult renal abnormalities in 60 to 80% [5, 6]. The spectrum of renal involvement in SSc is vast and ranges from asymptomatic renal abnormalities to hypertension, rise in serum creatinine level, proteinuria, and even life-threatening scleroderma renal crises (SRC) [6]. The prevalence of SRC is about 5–15%, with abrupt onset of hypertension and rapidly progressive renal failure due to vascular spasm and tissue ischemia. Certain risk factors, such as the use of corticosteroids, anti-RNA polymerase III, and diffuse skin involvement, increase the likelihood of developing SRC. In SSc patients with renal complaints, screening for SRC should be the first step, and if diagnosed, treatment with ACE inhibitors is recommended [6, 7]. In addition, studies have shown that SSc patients with renal involvement have poorer outcomes [8]. Renal involvement in SSc generally manifests with arterial hypertension with or without proteinuria. Although nephrotic syndrome has been reported in some SSc cases, it is rare and typically occurs due to amyloidosis or certain drugs rather than being directly linked to SSc [9, 10]. Herein, we report a case of diffuse SSc complicated with nephrotic syndrome (focal segmental glomerulosclerosis) due to its rarity and for a better outlook in the management of these cases.

## Case presentation

A female Caucasian patient from Gilan, Iran, aged 59 and housewife with a previous diagnosis of diffuse systemic sclerosis 8 years ago, came to our clinic complaining of anasarca and weight gain. She has no prior history of hypertension or diabetes. She was being treated regularly with multiple medications, including prednisolone 2.5 mg every other day, azathioprine 50 mg daily, and captopril 50 mg twice a day; the disease was under control with the regular use of her medications up until 1 week prior to her visit when she developed periorbital and bilateral lower limb edema, 4 kg weight gain, malaise, and decrease of appetite. She had no previous history aside from her SSc and no social history of alcohol use or smoking.

Examination revealed that the patient was conscious and oriented, with a regular heart rate of 82 beats/min. The respiratory rate was 14/min with an oxygen saturation of 100% in room air, and his blood pressure was 110/85 mmHg without significant differences between both arms. Lung sounds were clear, and the heart sounds were normal without any additional sounds or murmurs. Both lower extremities had four plus pitting edema, but all extremity pulses were detected. The patient also did not have any lymphadenopathy, and skin examination showed no petechia, purpura, or bruises. The examination was otherwise unremarkable.

The patient’s laboratory findings demonstrated a white blood cell count of 10.0 × 10^9^/L (ref: 4.4–11.0 × 10^9^/L), hemoglobin of 13 g/dl (ref: 14 to 17.5 g/dl), mean corpuscular volume of 94.6 fl (ref: 80–100 fl), platelet count of 204 × 10^9^/L (ref: 150–450 × 10^9^/L), creatinine of 1.9mg/dl and then 2.1 mg/dl (ref: 0.6–1.2 mg/dl), albumin of 1.5g/dL (ref: 3.6–5.2 g/dL), Estimated sedimentation rate (ESR) of 106 mm/hr (ref: 0–15 mm/h), Anti-nuclear antibody (ANA) of 1/2560, positive Anti SCL70, and normal C-reactive protein, Anti-glomerular basement membrane antibody, Anti-centromere antibody, C3, C4, CH50, IgG, IgA levels. Urine analysis demonstrated turbid colour urine, with microscopic hematuria (5–7 red blood cell count) and also red blood cell casts, while 24-h urine analysis demonstrated a volume of 2400cc, with 1356 mg creatinine, 30,960 mg protein, and 22,080 mg albumin (Table 1).

**Table 1:** Laborato﻿ry data of 59 years old female with a history of SSc who was diagnosed with nephrotic syndrome

Test (value)	Results	Reference value
White blood cell (× 10^9^/L)	10.0	4.4–11.0
Hemoglobin (g/dl)	13	14–17.5
Mean corpuscular volume (femtoliter)	94.6	80–100
Platelet count (× 10^9^/L)	204	150–450
Creatinine (mg/dl)	2.1	0.6–1.2
Albumin (g/dl)	1.5	3.6–5.2
ESR (mm/h)	106	0–15
ANA	1/2560	–
Anti SCL70	Positive	–
CRP (mg/l)	Normal	–
Anti-glomerular basement membrane antibody	Normal	–
Anti-centromere antibody	Normal	–
C3 (mg/dl)	Normal	–
C4 (mg/dl)	Normal	–
CH50	Normal	–
IgG	Normal	–
IgA	Normal	–
Urine analysis	Microscopic hematuria (5–7 red blood cell count) and red blood cell casts	–
24h urine volume (cc)	2400	800–2000
24h urine creatinine (mg/day)	1356	500–2000
24h urine protein(mg/day)	30,960	< 150
24h urine albumin (mg/day)	22,080	< 30

ANA: antinuclear antibody; CRP: c-reactive protein; ESR: Erythrocyte sedimentation rate; SCL: scleroderma; SSc: Systemic sclerosis

Chest X-ray and echocardiography were normal. Renal ultrasonography revealed normal-sized kidneys with normal cortico-medullary differentiation. Based on our initial assessment and laboratory findings, nephrotic syndrome was suspected and the patient was scheduled for a kidney biopsy.

**Fig. 1:**
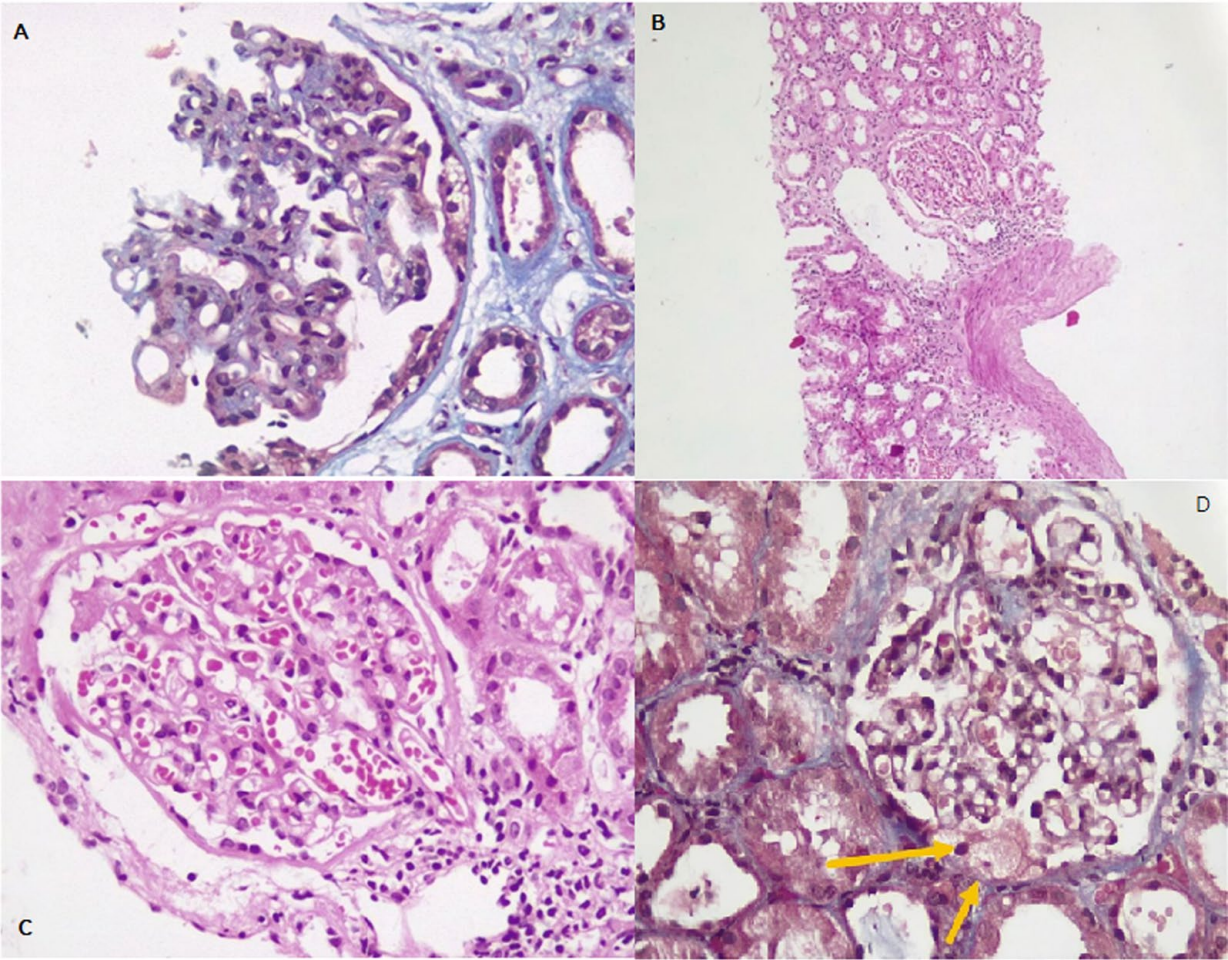
**A** A glomerulus with mild mesangial expansion, proliferation, and sclerosis. Rare foam cells are seen beneath the bowman capsule. Interstitial fibrosis is also evident adjacent to the glomerulus (Trichrome stain); **B** A glomerulus with mild mesangial proliferation, slight mononuclear leukocytic infiltration within interstitium is evident (H&E stain); **C** same glomerulus as **B**, with higher power showing mild mesangial proliferation (H&E stain); **D** Tiny focus of adhesion and rare foam cells deposition (arrows) at tip location (Trichrome stain)

Kidney biopsy revealed tiny focus of adhesion and foam cells deposition at the tip location, focal mild to moderate mesangial proliferation and focal global glomerular sclerosis and obsolescence and foci of mild acute tubular injury with IF/TA about 5% of cortex. These histopathological findings were subtle but compatible with tip variant of focal segmental glomerulosclerosis (Fig. 1).

For treatment of glomerulonephritis, the patient received 1 mg/kg/day of oral prednisolone while monitoring her blood pressure and blood glucose level, which was gradually tapered during a period of 6 months following remission. Alongside corticosteroid therapy, in the course of hospitalization, rituximab with a dosage of 1 gm was also started for the patient and repeated 2 weeks and 6 months later. After, in total, 3 doses of rituximab, improvement of the patient’s symptoms and laboratory data were observed. Lower limb edema was improved with 10 kg weight loss, proteinuria was decreased below 300 mg daily, and the serum creatinine level became normal (1.1 mg/dl).

## Discussion

In the present case, our patient had been diagnosed with diffuse SSc 8 years before developing nephrotic syndrome (focal segmental glomerulosclerosis). It is uncommon for patients with SSc to develop nephrotic syndrome, but certain drugs, such as D-penicillamine, can induce it, or rarely, it can be a manifestation of secondary amyloidosis [11]. To the best of our knowledge, our patient had not been prescribed any medication that leads to nephrotic syndrome; however, since the association between SSc and nephrotic syndrome is rare, it is essential to note that the results of this case do not necessarily prove that nephrotic syndrome is a consequence of SSc.

SSc is a condition that often affects the kidneys. Clinical manifestation of renal involvement occurs in 50% of the patients, but postmortem evaluation reveals occult renal pathology in 80% of patients with SSc [5]. Scleroderma renal crisis (SRC) is the most prominent renal involvement among systemic sclerosis patients, with a prevalence estimated to be between 5 and 15% [7]. SRC manifests with a sudden elevation of serum creatinine level, with or without hypertension [6]. Prior to the 1990s, SRC was the primary cause of death in SSc patients, but with the use of ACE inhibitors, mortality rates have significantly been reduced [7]. Isolated proteinuria is a marker of renal damage and vasculopathy in SSc patients. An increase in total protein excretion was observed in 17.5% of patients, and albuminuria was seen in 25% of patients with SSc. Proteinuria exceeding 1 g per day is uncommon in SSc and indicates glomerular diseases [6]. Another renal manifestation of SSc is an isolated decrease in GFR detected in 10% of patients with SSc [12]. A reduction in GFR is commonly associated with comorbid conditions accompanying SSc, such as hypertension and myocardial involvement [13]. The incidence of ANCA-associated vasculitis as renal involvement in patients with systemic sclerosis (SSc) is limited, with a 0–12% range. [14]. It is characterized by hypertension, proteinuria, and an increase in serum creatinine level; usually, a renal biopsy is required to confirm the diagnosis [5].

A few studies have reported the coexistence of SSc and nephrotic syndrome. In 2022, Abdulrasheed *et al*. reported a female case of 17 years old who initially presented with nephrotic syndrome (minimal change disease) and later, after seven months, developed diffuse SSc [10]. Hirohata *et al*. also reported a 39-year-old female with SSc who developed nephrotic syndrome (minimal change disease) four years later and was treated successfully with 60 mg of oral prednisolone daily without recurrence [9]. In yet another case in 2017, a 12-year-old female was diagnosed with nephrotic syndrome (FSGS) as the first manifestation of juvenile SSc [15]. In our case, the patient developed nephrotic syndrome (FSGS) eight years after being diagnosed with diffuse SSc.

In the general population, FSGS accounts for 20% and 40% of nephrotic syndrome cases in children and adults, respectively. Although rare, FSGS can be associated with scleroderma. It is histologically categorized into multiple variants, including not-otherwise-specified, perihilar, cellular, tip, and collapsing disease [16]. In our case, the kidney biopsy revealed a tip variant of FSGS in pathology, which has a better chance of complete remission compared to other variants [17]. Viruses, medication, and genes can cause FSGS. Diagnosis involves laboratory tests, clinical findings, and kidney biopsy [18]. Although a serum biomarker called urokinase-type plasminogen activator receptor (uPAR) is used to diagnose FSGS, its value is yet to be determined [19]. In our case, we confirmed the diagnosis based on pathological findings of the kidney biopsy. Treatment of FSGS typically involves steroid and immunosuppressive drugs. The duration of steroid therapy in treating FSGS is usually 16 weeks. However, immunosuppressive agents such as calcineurin inhibitors can be prescribed in cases where steroid is contraindicated or in cases with relapse. In some case reports and small case series, rituximab in steroid-sensitive FSGS patients demonstrated good therapeutic effects and improved survival rates [20, 21]. In the present case, with administration of rituximab alongside steroids, we achieved complete remission of the nephrotic syndrome in our patient.

During the treatment of patients with SSc, it is crucial to proceed with caution when administering high doses of corticosteroids due to the risk of SRC, which is one of the leading causes of mortality in SSc patients. To ensure patient safety, it is essential to rule out SRC in every patient with SSc experiencing renal complications when administering steroids. Renal crisis results in an increase in blood pressure in 90% of cases and a decrease in GFR. Although SRC can occur even with normal blood pressure, laboratory tests may reveal hemolytic microangiopathic anemia [6]. In our case, blood pressure was normal, and in laboratory evaluation, we found no evidence of hemolysis or any drop in hemoglobin level nor schistocyte in peripheral blood smear. Hence, we were assured that high doses of steroids administered during her treatment did not result in SRC.

The limitation of this case was the unavailability of a uPAR biomarker for the diagnosis of FSGS due to our country’s burden.

## Conclusion

SSc is a complex disease that impacts various systems within the body. The most important type of renal involvement in SSc is SRC. However, other renal pathologies can also occur. Although nephrotic syndrome is a rare renal involvement associated with SSc, we reported a case of FSGS in a patient with SSc here.

1. It is crucial to explore other potential kidney pathologies when a SSc patients exhibit a different clinical picture.
2. A renal biopsy is essential, if a patient with SSc has sustained proteinuria or nephrotic range proteinuria to establish a diagnosis.
3. Each patient must undergo a comprehensive investigation, as the treatment for SRC and other renal pathologies, including nephrotic syndrome, differs significantly.

## Data Availability

All data regarding this case has been reported in the manuscript. Please contact the corresponding author if you are interested in any further information.
